# An overview of key pretreatment processes employed for bioconversion of lignocellulosic biomass into biofuels and value added products

**DOI:** 10.1007/s13205-013-0167-8

**Published:** 2013-09-05

**Authors:** Venkatesh Chaturvedi, Pradeep Verma

**Affiliations:** 1 School of Biotechnology, Banaras Hindu University, Varanasi, Uttar Pradesh India; 2 Department of Biotechnology, Guru Ghasidas Vishwavidyalaya, Bilaspur, Chhattisgarh India; 3 Present Address: Department of Microbiology, Central University of Rajasthan, N.H. 8 Bandarsindri, Kishangarh, Ajmer, Rajasthan India

**Keywords:** Pretreatment, Biomass, Lignin, Cellulose, Biofuel

## Abstract

The hunt for alternative sources of energy generation that are inexpensive, ecofriendly, renewable and can replace fossil fuels is on, owing to the increasing demands of energy. One approach in this direction is the conversion of plant residues into biofuels wherein lignocellulose, which forms the structural framework of plants consisting of cellulose, hemicellulose and lignin, is first broken down and hydrolyzed into simple fermentable sugars, which upon fermentation form biofuels such as ethanol. A major bottleneck is to disarray lignin which is present as a protective covering and makes cellulose and hemicellulose recalcitrant to enzymatic hydrolysis. A number of biomass deconstruction or pretreatment processes (physical, chemical and biological) have been used to break the structural framework of plants and depolymerize lignin. This review surveys and discusses some major pretreatment processes pertaining to the pretreatment of plant biomass, which are used for the production of biofuels and other value added products. The emphasis is given on processes that provide maximum amount of sugars, which are subsequently used for the production of biofuels.

## Introduction

**Fig. 1:**
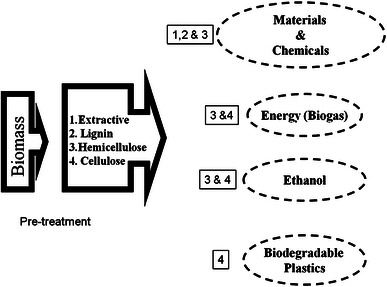
Schematic representation of steps involved in the conversion of plant biomass into biofuels and other value added products (Alvira et al. 2010)

In recent years, due to the world’s energy demand and with the uncertainty in the supply, ever increasing prices of fossil fuels, causing increased environmental pollution, it has become imperative to scout alternative sources of energy which can replace conventional fossil fuels (Wan and Li 2011). In this context, the conversion of plant biomass/products into biofuels and biochemicals has gained impetus due to the feasibility of an alternative processes available to convert the complex biomass into biofuels and biomaterials (Adsul et al. 2011). As a result, the lignocellulosic materials become again an important resource to provide and support a sustainable and environmentally friendly system (Itziar et al. 2012). Figure 1 summarizes the conversion of plant biomass into biofuels and other value added products.

**Fig. 2:**
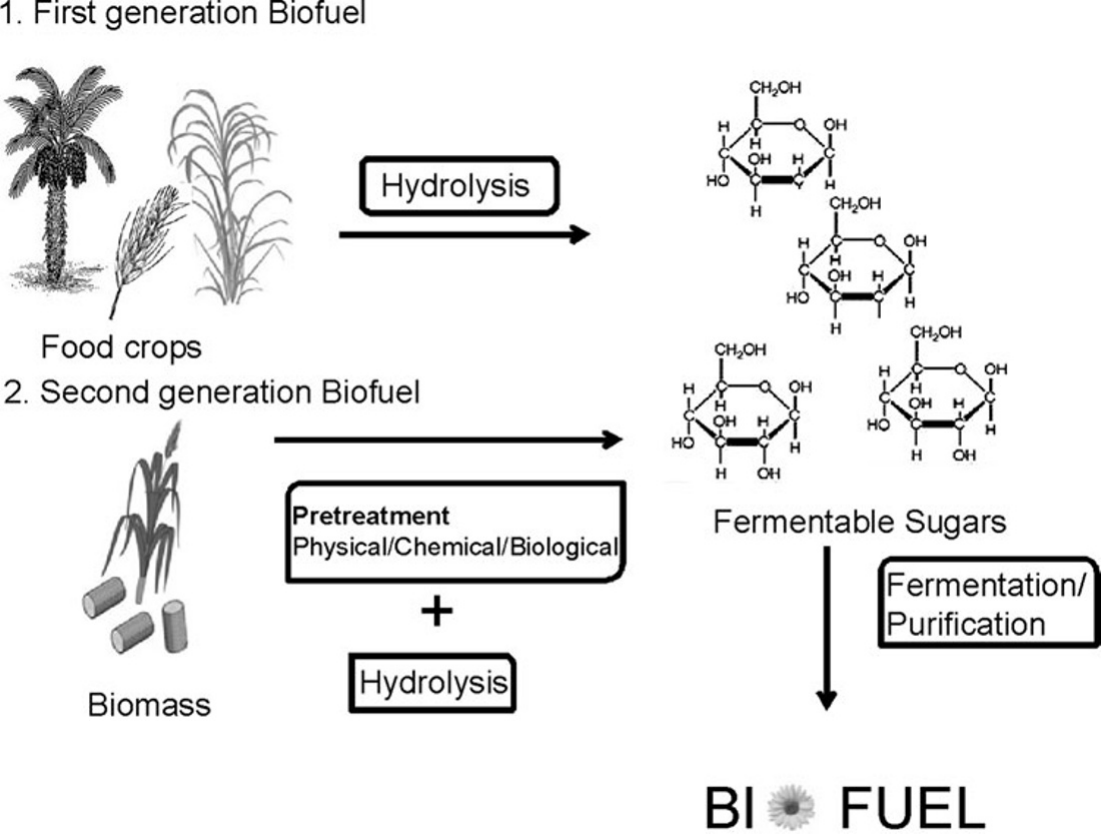
Steps involved in the synthesis of the first- and second-generation biofuels (Sun and Cheng 2002)

Biofuels can be broadly categorized as first- and second-generation biofuels. Biofuels which are derived from starch, sugars, and plant oils are termed as first-generation biofuels. These include bioethanol derived from food crops such as sugar beet, cereals, sugarcane and biodiesel obtained from rape, sunflower, soya, and palm, respectively (Ghose et al. 1983). Biofuels derived from lignocellulosic biomass such as hard/soft wood, agricultural wastes are called as second-generation biofuels (Sun and Cheng 2002) (Fig. 2).

Among these, the production of the researches in the direction of second-generation biofuels is in stride due to easy availability of lignocellulosic biomass (Sánchez and Cardona 2008). According to National Renewable Energy Laboratory (NREL), in 2010, production of ethanol in the USA was around 5 billion gallons/annum and was contributed by food grains. With the advent of second-generation biofuels, it is expected that in 2015, the production will rise to 8 billion gallons/annum and a major portion will be contributed by lignocellulosic biomass.

A major portion of this biomass comprises cellulose, hemicelluloses, lignin and pectin. Among these constituents, cellulose and hemicelluloses are polymers of sugars and can be hydrolyzed to form fermentable sugars. Lignin forms a protective covering, which protects cellulose and hemicelluloses from degradation by various fungi and bacteria. Presence of lignin impedes enzymatic hydrolysis of cellulose and hemicellulose. Therefore, a pretreatment processing step is required for depolymerization of lignin before conversion of cellulose and hemicellulose into simple sugars (Balan et al. 2009; Adsul et al. 2011).

**Fig. 3:**
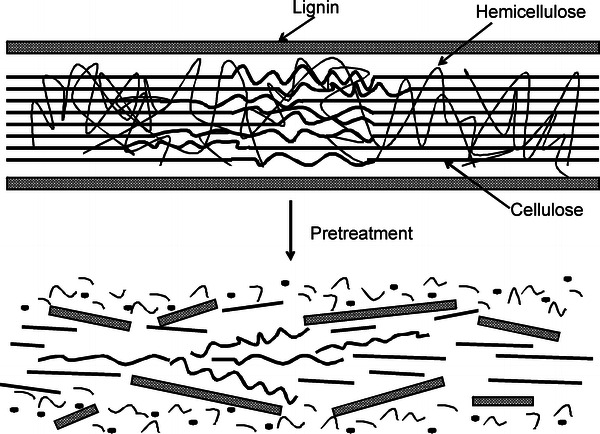
Effects of various pretreatment processes on structure of lignocellulose (adapted from Hsu et al. 1980)

A number of pretreatment technologies based on numerous physical, chemical and biological methods have been developed, which alter/damage the structure of lignocelluloses and remove lignin (Alvira et al. 2010; Geddes et al. 2011). The exposed complex carbohydrates such as cellulose and hemicelluloses are then hydrolyzed to form fermentable sugars (Fig. 3). An effective pretreatment process should have following advantages: it should preserve and decrystallize the celluloses, depolymerize hemicelluloses; it should restrict the formation of inhibitors which resist the hydrolysis of carbohydrates; it should have low energy input, recovery of value added products such as lignin; and at last it should be cost effective (Agbor et al. 2011).

In this review, we have emphasized on some of the major and widely used chemical, and biological pretreatment processes which are employed for treatment of different lignocellulosic biomass aiming at removal of lignin and conversion of cellulose and hemicellulose into reducing sugars, which can be further employed for the production of bioethanol or other value added products. We have discussed the main principles behind these pretreatment processes, their mechanisms, merits/demerits and maximum yield of sugars which is obtained. The processes are as follows.

## Pretreatment of biomass under acidic conditions

This process involves the treatment of lignocellulosic biomass with different acids such as sulfuric acid, oxalic acid, peracetic acid, respectively. Dilute acid treatment is considered as a cheap and effective pretreatment method due to low cost and easy availability of acids (Kim et al. 2005). Acid treatment is carried out in the presence of high and low concentrations of acids, and at high and low temperatures. At high temperatures, pretreatment of biomass is performed at temperature greater than 160 °C in continuous process containing a low concentration of biomass (5–10 % substrate concentration). In general, dilute sulfuric acid is sprayed on raw biomass and incubated at 160–220 °C for few minutes (González et al. 1986). At high temperature (160–220 °C), hemicellulose gets hydrolyzed, releasing monomeric sugars and soluble oligomers from the cell wall matrix. Hemicellulose removal increases the porosity and improves enzymatic digestibility of cellulose (Kim et al. 2005). During dilute acid treatment, hemicellulose is hydrolyzed, whereas cellulose and lignin are unaffected. The major pit hole of such process is the synthesis of furfural and hydroxymethyl furfural, which are produced by dehydration of hexoses and pentoses, respectively (Liu and Song 2009). These are considered as a strong inhibitor in microbial fermentation. While at low temperatures, the process is carried out at temperature below 160 °C in batch process at high biomass concentration (10–40 % of substrate). In this process, concentrated strong acids such as H_2_SO_4_ and HCl are widely used for treating lignocellulosic materials because they are powerful agents for cellulose hydrolysis (Sun and Cheng 2002). In the presence of concentrated acids, hemicellulose and cellulose get hydrolyzed completely, whereas lignin is unaffected. The advantage of using concentrated acids in the pretreatment process is the high yield of monomeric sugar, but the major drawback of concentrated acid is their corrosive nature and the need to recycle acids to lower the cost of pretreatment.

Hog fuel and pin chips are byproducts produced by sawmills. A mixture of Hemlock hog fuel/pin chips (85:15 ratio) was pretreated by a two-stage dilute sulfuric acid treatment (Kim et al. 2005). The optimum conditions in this two stage treatment regime were; in the first stage biomass was treated with 1.1 % acid at  190 °C for 150 s and in the second stage biomass was treated with 2.5 % acid at 210 °C for 115 s respectively. Results indicated that at these pretreatment conditions, total sugar yield of 26.9 % was obtained. Similarly, olive tree biomass consisting of thin branches and leaves was pretreated with dilute sulfuric acid (Cara et al. 2008). Pretreatment was performed in the presence of varying acid concentration (0.2–1.4 %) and at different temperatures (170–210 °C). Highest sugar yield (48.6 %) was observed at 170 °C and 1.0 % acid concentration. Biomass (5–20 wt%), sulfuric acid (0.5–1.5 vol. %) and temperature at 120 and 140 °C were varied using dilute sulfuric acid treatment of corn fibers by Noureddini and Byun (2010). They observed that maximum yield of reducing sugars (56.8 %) was obtained at 0.5 % sulfuric acid, 5 % biomass and at 140 °C. Furfural formation was low as compared to other conditions. Similarly, sugarcane tops were treated with dilute acid treatment under optimized conditions for ethanol production by Sindhu et al. (2011). Maximum (68.5 %) sugar yield was obtained in the presence of 25 % w/w biomass, 3 % sulfuric acid for 60 min. Apart from sulfuric acid, many workers have employed other acids such as Peracetic acid (PAA) and oxalic acid for the treatment of biomass. Corn cobs were pretreated with oxalic acid (Lee et al. 2011). In their study, Corncob pellets were impregnated with oxalic acid. The pellets were rapidly heated to 168 °C and held for 26 min. Results indicated that in oxalic acid treatment, a total sugar yield of 13.1 % was obtained. The treated biomass also contained a low level of inhibitors. Further, Lee and Jeffries (2011) compared the effect of maleic, oxalic and sulfuric acid on the treatment of corn cob. Their results clearly indicated that in the presence of maleic acid, highest yield of reducing sugars 10 % was obtained. The treatment resulted in higher levels of furfural and hydroxymethyl furfural formation. Many workers have evaluated the combination of two pretreatment processes for biomass. Kim et al. (2011) studied pretreatment of rice straw in a two-stage pretreatment process using aqueous ammonia and dilute sulfuric acid in percolation mode. Under optimum conditions, the yield of reducing sugars in both the stages was 96.9 and 90.8 %, respectively. Their study clearly indicated that combination of these two processes resulted in better removal of lignin and hemicellulose from rice straw. Similar to this study, pretreatment of *Eulaliopsis binata*, a perennial grass commonly found in India and China, was performed using dilute sulfuric acid (Tang et al. 2013). The optimum conditions for the pretreatment were 0.5 % dilute sulfuric acid, 160 °C for 30 min, and at a solid-to-liquor ratio of 1:5. The results showed that the pretreatment liquor contained 21.02 % total sugars, 3.22 % lignin and 3.34 % acetic acid and low levels of inhibitors. Steam pretreatment of corn stover in combination with sulfuric acid was studied by Bondesson et al. (2013). The highest yield using sulfuric acid (78 %) was achieved using pretreatment at 200 °C for 10 min. In order to obtain maximum sugar yield, the synergetic effect of mild acid and alkali with Electron Beam Irradiation (EBI) on the enzymatic hydrolysis of grass was evaluated by Karthika et al. (2012). In their experimental setup, grass samples were soaked with 1 % H_2_SO_4_/1 % NaOH, exposed to 75 and 150 kGy of EBI. Water-presoaked biomass was used as control. Hydrolysis of pretreated samples was carried out using cellulase (15 FPU/g biomass) for 120 h. Reducing sugar and glucose yields from enzymatic hydrolysis were 32 % in NaOH treated and exposed to 75 kGy, and 43 % in NaOH treated and 150 kGy treated, respectively. The yields were significantly higher when compared with untreated control (19 %). Removal of hemicellulose, decreased crystallinity and structural changes were major factors for the combined treatment effect favoring the hydrolysis. This survey clearly indicates that only acid treatment is insufficient for higher sugar yields and thus combination of two different pretreatment processes, such as the study conducted by Kim et al. (2011), where rice husk was pretreated in two stages with two pretreatment processes and very high yield of reducing sugars (90.8 %) was obtained.

Acid pretreatment of biomass is inexpensive because acids such as sulfuric acid and hydrochloric acid employed are cheap. The process is carried out at high temperatures, and therefore, it requires high energy input, which is costly. Secondly, presence of acids at high temperatures can be corrosive, and thus, the process requires specific reaction vessels which must be resistant to these conditions. In addition, acid treatment generates inhibitors which have to be removed and as a result downstream processing costs are high.

## Pretreatment of biomass under alkaline conditions

In the alkaline treatment, biomass is treated with alkali such as sodium, potassium, calcium and ammonium hydroxides at normal temperature and pressures. The main advantage of the process is efficient removal of lignin from the biomass. Many reports have demonstrated that the process removes acetyl and uronic acid groups present on hemicellulose and thus enhances the accessibility of the enzyme that degrades hemicellulose (Chang and Holtzapple 2000). In the alkali treatment process, ester linkages that join xylan and hemicelluloses residues are also hydrolyzed (Sun and Cheng 2002). Alkaline treatment is also divided into two types on the basis of alkali employed. These include:

### Pretreatment with calcium, sodium and potassium hydroxide

Lime (calcium hydroxide) or sodium hydroxide is most commonly employed. The reaction conditions are generally mild, which prevent condensation of lignin leading to its high solubility and greater removal. In these conditions, degradation of sugars is minimal (Sharma et al. 2012). Such process is usually applied to biomass having low lignin content such as grasses and softwood. Addition of air or oxygen has shown to significantly enhance the delignification process (Chang and Holtzapple 2000). Lime pretreatment of corn stover was studied by Kim and Holtzapple (2005). Corn stover was pretreated with an excess of calcium hydroxide (0.5 g Ca(OH)_2_/g raw biomass) in non-oxidative and oxidative conditions at 55 °C for 4 weeks with aeration. Under these conditions, the total yield of glucose and xylose was 91.3 and 51.8 % at 15 FPU/g cellulose, and 95.5 and 53.5 % at 60 FPU/g cellulose, respectively. Similarly, lime pretreatment of rice hulls for the production of ethanol was studied by Saha and Cotta (2008). In their study, milled rice hulls (15.0 % w/v) and lime at different concentrations were mixed in water, and autoclaved at 121 °C for 1 h. Results indicated that the maximum yield of total sugars (12.6 %) was achieved at 100 mg lime/g hulls and 1 h pretreatment time. Also, lime pretreatment did not generate furfural and hydroxymethyl furfural in the hydrolysate. Many workers have evaluated the effect of sodium hydroxide on different biomasses. Zhao et al. (2008) studied alkaline pretreatment using sodium hydroxide of spruce at low temperature in both presence and absence of urea. It was observed that the enzymatic hydrolysis rate and efficiency were significantly improved by treatment process. Results showed that after the treatment process, lignin was slightly removed; however, the structure was altered that cellulose was accessible to hydrolytic enzymes and thus enzymatic hydrolysis greatly improved after the treatment process. Optimization of the process leads to 70 % glucose yield at low temperature (−15 °C) using 7 % NaOH/12 % urea solution. The optimum conditions for pretreatment was found to be 3 % NaOH/12 % urea and −15 °C and around 60 % glucose conversion was achieved. Similar to this study, effects of potassium hydroxide (KOH) treatment on switch grass was studied by Sharma et al. (2012). Results indicated that about 99.26 % sugar retention was observed at 0.5 % KOH, at 21 °C and after 12 h treatment. Further, mentioned treatment caused production of 58.2 % of reducing sugar. Many workers have used combination of two pretreatment processes. Combination of alkaline treatment (lime) with oxidative delignification process for treatment of Alamo switch grass (*Panicum virgatum*) was studied by Falls and Holtzapple (2011). Optimum conditions were found to be 110 °C, 6.89 bar O_2_, 240 min, 0.248 g Ca(OH)_2_/g biomass. Results indicated that 2.6 % glucan and significant levels of reducing sugars (26.0 %) in raw biomass were recovered from the pretreatment liquor. Pretreatment of *Miscanthus* sp. biomass using two-stage dilute sulfuric acid and lime pretreatment was studied by Guo et al. (2013). The optimized conditions using response surface methodology were, 0.73 wt% H_2_SO_4_, 150 °C, 6.1 min in the first stage, and 0.024 g lime/g biomass, 202 °C, 30 min in the second stage showed more than 80 % recovery of glucose and more than 70 % recovery of xylose.

The present survey clearly suggests that alkaline pretreatment process involving calcium, sodium and potassium hydroxides gave higher yields of reducing sugars with biomass having low lignin content, such as rice hulls and grasses. Although, lime and other hydroxides are inexpensive, down stream processing costs are high, thus making it a costly process. The process utilizes a huge amount of water for washing salts of calcium and sodium, which are incorporated into the biomass and are difficult to remove (González et al. 1986). In addition, the process also generates inhibitors during depolymerization of lignin which have to be removed prior to hydrolysis step.

### Pretreatment with ammonia

The process involves aqueous ammonia treatment at elevated temperatures. The process sufficiently reduces lignin content and removes some hemicellulose, while cellulose is decrystallized. Ammonia pretreatment techniques can be divided into three broad types, viz., the Ammonia Recycle Percolation (ARP) (Kim et al. 2003), Ammonia Fiber Explosion method (AFEX) (Teymouri et al. 2005) and Soaking in Aqueous Ammonia (SAA) treatments (Kim et al. 2008), respectively. In ARP treatment, the biomass is pretreated with aqueous ammonia in a flow-through column reactor. The liquid flows at high temperature through the reactor column, which is packed with biomass (Kim et al. 2003; Kim and Lee 2005). Incubation in the presence of ammonia leads to hydrolysis of hemicellulose and destruction of lignin by ammonolysis. In this process, cellulose forms a complex with ammonia resulting in breaking of hydrogen bonds in cellulose. This leads to the loss of crystallinity of cellulose due to, which it becomes accessible to enzymatic hydrolysis (Schuerch 1963; Mittal et al. 2011). After the reaction, the solid fraction, rich in cellulose and hemicellulose, is separated from the liquid. The liquid fraction is sent into a steam-heated evaporator for ammonia recovery and lignin and other sugar separation. Ammonia is then recycled to the reactor inlet, whereas the separated fraction is sent into a crystallizer. After crystallization, a washing step is carried out to extract the sugars that have been retained in the solid matrix. Pretreatment of corn stover by ARP was evaluated by Kim and Lee (2005), at optimized conditions consisting of 0.47 g of NH_3_ and 2.7 g of water at 170 °C for pretreatment of 1 g of corn stover. Results indicated that 73.4 % delignification and 88.5 % digestibility with enzyme loading of 15 FPU/g of glucan were observed.

Soaking in Aqueous Ammonia (SAA) at low temperature removes lignin efficiently in the raw material by minimizing the interaction with hemicellulose. As a result an increase of surface area, pore size increases and retained hemicellulose/cellulose can be hydrolyzed to fermentable sugars by commercial enzymes. Pretreatment of corn stover in a two-stage percolation process consisting of hot water treatment followed by treatment with aqueous ammonia was evaluated by Kim and Lee (2006). The optimum operating conditions of the two-stage process in the first stage were: 190 °C, 5.0 mL/min, 30 min for hot water treatment and 170 °C, 5.0 mL/min, 60 min for aqueous ammonia treatment. Under these conditions, 92 % of xylan was hydrolyzed, of which 83 % was recovered, and 75 % of delignification was achieved. The treated biomass contained 78 % glucan, 3.6 % xylan and 9.8 % Klason lignin. The digestibility of hot water–ARP treated sample was 94 % with 60 FPU/g of glucan and 85 % with 15 FPU/g of glucan. Kim et al. (2008) evaluated SAA as a pretreatment method for barley hull. In their study, destarched barley hull was treated with 15–30 % aqueous ammonia, at 30–75 °C for 12 h to 77 days with no agitation and a solid-to-liquid ratio of 1:12. After soaking, the solids were recovered by filtrating, washed and analyzed. About 66 % of lignin was solubilized, and 83 % of glucan, 63 % of xylan were saccharified.

### Pretreatment with ammonia fiber explosion process

AFEX is considered a potential technique for pretreatment of lignocellulosic material (Holtzapple et al. 1992). In the AFEX process, biomass is treated with liquid anhydrous ammonia at temperatures (60–100 °C) and high pressure (250–300 psi) for 5 min. Then the pressure was released rapidly. The combined effect of ammonia and high pressure leads to swelling of lignocellulose biomass, disruption of lignocellulose architecture leading to hemicellulose hydrolysis, and decrystallization of cellulose. However, lignin remains unaffected during the process. Results have demonstrated that cellulose and hemicelluloses obtained by the process can be completely converted to fermentable sugars (Mosier et al. 2005). The biomass recovered using this process is dry as water is not used. The biomass is stable for long periods and no degradation of cellulose and hemicelluloses is observed. The main advantage of the process is that nearly all the ammonia which is employed can be recovered and reused. AFEX can be employed for various lignocellulosic materials, including rice straw, municipal solid waste, newspaper, sugar beet pulp, sugar cane bagasse, corn stover, switch grass, etc. Herbaceous crops and agricultural residues are well suited for AFEX. Only drawback of this process is that it cannot be employed for treatment of biomass having high lignin content such as hardwood and newspaper.

Pretreatment of growth and seed stage of reed canary grass by Ammonia Fiber Expansion (AFEX) followed by enzymatic hydrolysis using 15 Filter Paper Units (FPU) cellulase/g of glucan was studied by Bradshaw et al. (2007). Optimum conditions for pretreatment were: for vegetative growth stage of reed canary grass: 100 °C, 60 % moisture content, 1.2:1 kg ammonia/kg of dry matter and for seed stage of reed canary grass: 100 °C, 60 % moisture content, 0.8:1 kg ammonia/kg of dry matter. Maximum yield of reducing sugars after 168 h hydrolysis using 15 FPU Spezyme CP cellulase/g of glucan were 86 % glucose and 78 % xylose for vegetative growth stage of reed canary grass, and 89 % glucose and 81 % xylose for seed stage of reed canary grass. In addition, pretreatment of Empty Palm Fruit Bunch Fiber (EPFBF), generated from palm processing industry was investigated by Lau et al. (2010). Pretreatment was carried out using AFEX and enzymatic hydrolysis. The optimum conditions were at 135 °C, 45 min retention time, water to dry biomass loading of 1:1 (weight ratio), and ammonia to dry biomass loading of 1:1 (weight ratio). The particle size of the pretreated biomass was reduced post-AFEX. The optimized enzyme formulation consisting of Accellerase (84 μL/g biomass), Multifect Xylanase (31 μL/g biomass), and Multifect Pectinase (24 μL/g biomass) gave yield of 90 % of the total reducing sugars after 72 h of enzymatic hydrolysis. Shao et al. (2010) have demonstrated that AFEX-pretreated corn grain gave 1.5- to 3-fold higher enzymatic hydrolysis yield compared with the untreated substrates. Sequential addition of cellulases after hydrolysis of starch resulted in 15–20 % higher hydrolysis yield compared with simultaneous addition of hydrolytic enzymes. AFEX-pretreated corn stover gave 70 % glucan conversion after 72 h of hydrolysis for 6 % glucan loading (at 8 mg total enzyme loading per gram of glucan). Microbial inoculation of corn stover before ensilation yielded a 10–15 % lower glucose hydrolysis yield for the pretreated substrate, due to the loss in starch content. Ethanol fermentation of AFEX-treated (at 6 % w/w glucan loading) corn stover gave 93 % ethanol yield.

Pretreatment with ammonia has been proved to be an efficient method for biomasses having low lignin content. Among various alkaline pretreatment processes discussed, the highest yield of reducing sugars (80–90 %) is obtained in this process (Bradshaw et al. 2007). This process has shown high sugar yields and high percent removal of lignin from biomass. Pretreatment of biomass by employing ammonia in processes such as SAA, ARP, AFEX has shown promising results in terms of depolymerization of lignin. A major drawback of using ammonia is the cost involved in pretreatment process, which is very high. Recycling of ammonia during pretreatment has shown to decrease the operating cost. Another important concern while using ammonia is the environmental concerns that are related with ammonia usage. Since ammonia is toxic to the environment, suitable strategy has to be maintained to prevent its leakage in the environment. This additional feature also increases the cost of the process.

## Pretreatment of biomass using organosolv process

Organosolv pretreatment process involves extraction of lignin from plant biomass by employing organic solvent or mixture of solvents in combination with water. Solvents which are most commonly employed are ethanol, methanol, acetone, and ethylene glycol (Ichwan and Son 2011). Temperatures used in the process can be as high as 200 °C, but lower temperatures can be sufficient depending on the type of biomass and the use of a catalyst (Ghose et al. 1983). Possible catalysts include inorganic or organic acids (Sun and Cheng 2002). Organosolv extraction process causes hydrolysis of the internal bonds in lignin and also between lignin and hemicellulose. The organic solvents also cause hydrolysis of the glycosidic bonds in hemicelluloses and to a smaller extent in cellulose. The presence of acids as a catalyst causes acid-catalyzed degradation of the monosaccharides into furfural and 5-hydroxymethyl furfural followed by condensation reactions between lignin and these reactive aldehydes (Chum et al. 1985). After removal of lignin, the biomass (rich in cellulose) is used for enzymatic hydrolysis (Zhao et al. 2009).

Pretreatment of wheat straw by glycerol-based autocatalytic organosolv pretreatment was studied by Sun and Chen (2008). At optimized conditions corresponding to liquid–solid ratio of 20 g/g at 220 °C for 3 h, 70 % hemicelluloses and 65 % lignin were removed from the biomass. Also, 98 % cellulose retention was achieved. The pretreated fibers showed 90 % of the enzymatic hydrolysis yield after 48 h of incubation. Organosolv pretreatment of Horticultural Waste (HW) for bioethanol production was studied by Geng et al. (2012). A modified organosolv method using ethanol under mild conditions followed by H_2_O_2_ post-treatment was investigated for HW. Result showed that enzymatic hydrolysis of the organosolv pretreated HW with 17.5 % solid content, enzyme loading of 20 FPU/g HW of filter paper cellulase, and 80 CBU/g HW of β-glycosidase resulted in a HW hydrolysate containing 15.4 % reducing sugar after 72 h. Fermentation of the above hydrolysate medium produced 11.69 g/L ethanol at 8 h using *Saccharomyces cerevisiae*. Hideno et al. (2013) have shown that application of alcohol-based organosolv treatment in combination with Ball Milling (BM) to Japanese cypress (*Chamaecyparis obtusa*) greatly improved the enzymatic digestibility and decreased the required severity of organosolv treatment. Moreover, alcohol-based organosolv treatment increased the efficiency and reduced the time required for BM despite small quantity of removed lignin. It was found that the combination of alcohol-based organosolv treatment in mild condition and short-time BM had a synergistic effect on the enzymatic digestibility of Japanese cypress.

Ichwan and Son (2011) studied organosolv extraction of oil palm pulp using various solvents such as ethylene glycol–water, ethanol–water and acetic acid–water mixture to extract cellulose. The yield of organosolv pulping with ethylene glycol–water, ethanol–water and acetic acid–water mixture was 50.1; 48.1 and 41.7 %, respectively, while the kappa number was 74.7; 72.7; and 67. Fourier Transform Infrared Spectroscopy (FTIR) spectra showed the degradation of cellulose pulp occurred during acid pulping. The X-ray Diffraction (XRD) measurement showed that the ethanol–water mixture pulping resulted in higher crystallinity of pulp (68.67), followed by ethylene glycol–water mixture as (58.14), acid pulping (54.21). Further Panagiotopoulos et al. (2012) performed pretreatment approach, employing steam followed by organosolv treatment to fractionate hemicellulose, lignin and cellulose components from poplar wood chips. The results indicated that lignin extraction was enhanced as more than 66 % of lignin was extracted. More than 98 % of the original cellulose was recovered after the two-stage pretreatment and 88 % of the cellulose could be hydrolyzed to glucose at enzyme loading of 5 FPU/g of cellulose after 72 h. Organosolv extraction of sugarcane bagasse was evaluated by Mesa et al. (2011). Under optimized conditions, 30 % (v/v) ethanol at 195 °C for 60 min produced 29.1 % of reducing sugars. The scale-up of the process, by performing the acid pretreatment in a 10-L semi-pilot reactor fed with direct steam, was successfully performed. Recently, Pan and Saddler (2013) employed extracted lignin obtained from ethanol organosolv and used it as a replacement of petroleum-based polyol for the production of Rigid Polyurethane Foams (RPFs). The results suggested that the prepared foams contained 12–36 % (w/w) extracted lignin. The density, compressive strength, and cellular structure of the prepared foams were investigated and compared and it was found that the lignin-containing foams had comparable structure and strength with polyol containing foams.

Organosolv process has been extensively utilized for extraction of high-quality lignin, which is a value added product, and once the lignin is removed from the biomass, the cellulose fibers become accessible to enzymes for hydrolysis and absorption of cellulolytic enzymes to lignin is minimized, which leads to higher conversion of biomass. According to the present survey, this process has shown high amounts of enzymatic hydrolysis of treated biomass (around 90 %) due to efficient removal of lignin. The main drawback of the process is that the solvent and the catalysts that are employed are costly. Removal and recovery of the solvent can considerably reduce the operational cost (Sun and Cheng 2002). Another important aspect is safety measures which have to be implemented because organic solvents are inflammable and uncontrolled use can cause fires and explosions. This additional requirement increases the cost of the process. Organic solvents are inhibitors of enzymatic hydrolysis, so their removal is necessary for proper enzymatic hydrolysis of biomass (Mosier et al. 2005; Agbor et al. 2011). Removal of organic solvents also poses an additional cost on the process.

## Pretreatment of biomass by oxidative delignification

Delignification of lignocellulose can also be achieved by treatment with an oxidizing agent such as hydrogen peroxide, ozone, oxygen or air (Hammel et al. 2002; Nakamura et al. 2004). In such process, lignin is converted to acids. However, these acids can act as inhibitors during fermentation process. Therefore, acids have to be removed (Alvira et al. 2010). In addition, oxidative treatment also damages the hemicellulose fraction of the lignocellulose complex and a major portion of the hemicellulose gets degraded and becomes unavailable for fermentation (Lucas et al. 2012). The major types of oxidative delignification process are as follows.

### Pretreatment by hydrogen peroxide

Hydrogen peroxide is the most commonly employed oxidizing agent. Studies have shown that dissolution of about 50 % of lignin and most of the hemicellulose has been achieved in a solution of 2 % H_2_O_2_ at 30 °C. Studies have shown that hydrolysis of hydrogen peroxide leads to the generation of hydroxyl radicals, which degrade lignin and produce low molecular weight products. Removal of lignin from lignocellulose leads to the exposure of cellulose and hemicellulose causing increased enzymatic hydrolysis (Hammel et al. 2002). The yield of enzymatic hydrolysis followed can be as high as 95 %. Combined chemical (H_2_O_2_) and biological treatment of rice hull was performed by Yu et al. (2009). Their study consisted of chemical treatment (H_2_O_2_) followed by biological treatment with *Pleurotus ostreatus*. The combined pretreatments led to significant increases of the lignin degradation. At optimum conditions, H_2_O_2_ (2 %, 48 h) and *P. ostreatus* (18 days) led to 39.8 and 49.6 % of net yields of total sugar and glucose, respectively. It was about 5.8 times and 6.5 times more than that of the sole fungal pretreatment (18 days), and it was also slightly higher than that of the sole fungal pretreatment for 60 days. A study aimed at increasing the enzymatic digestibility of sweet sorghum bagasse for bioethanol production was performed by Cao et al. (2012). Among different chemical pretreatment methods employed, the best yields were obtained when sweet sorghum bagasse was treated with dilute NaOH solution followed by autoclaving and H_2_O_2_ immersing pretreatment. The highest cellulose hydrolysis yield, total sugar yield and ethanol concentration were 74.3, 90.9 % and 6.1 g/L, respectively, which were 5.9, 9.5 and 19.1 times higher than the control. Lucas et al. (2012) studied the effect of manganese acetate as a catalyst in oxidative delignification of wood with hydrogen peroxide at room temperature. When exposed to a mixture of hydrogen peroxide and manganese acetate in aqueous solution, poplar wood sections were converted into a fine powder-like material. Optical and Raman microscopy showed oxidation of lignin containing middle lamellae. Raman spectra from the solid residue revealed a delignified and cellulose-rich material. Glucose yields following enzymatic hydrolysis were 20–40 % higher in poplar sawdust pretreated with hydrogen peroxide and manganese acetate for 2, 4, and 7 days at room temperature than those in sawdust exposed to water only for identical durations, suggesting the viability of the mild, inexpensive method for pretreatment of lignocellulosic biomass. Alkaline Hydrogen Peroxide (AHP) pretreatment of Cashew Apple Bagasse (CAB) was evaluated by Correia et al. (2013). In their study, the effects of the concentration of hydrogen peroxide at pH 11.5, the biomass loading and the pretreatment duration performed at 35 °C and 250 rpm were evaluated after the subsequent enzymatic saccharification of the pretreated biomass using a commercial cellulase enzyme. At optimized conditions, consisting of solid loading of 5 % (w/v) at 4.3 % AHP, 6 h, 35 °C, a total reducing sugar yield of 42.9 % was obtained.

Pretreatment with hydrogen peroxide is considered a very harsh treatment which leads to lignin removal and high yields of reducing sugars. Reducing sugar yields up to 90 % have been obtained (Cao et al. 2012). A major drawback of this process is that it is costly owing to high cost of hydrogen peroxide and requirement of reactions vessels that can withstand such conditions. Another drawback of the process is the generation of inhibitors, which are needed to be removed prior to enzymatic hydrolysis. This additional step also increases the cost significantly.

### Pretreatment by ozonolysis

In the ozonolysis process, the biomass is treated with ozone, which causes degradation of lignin by attacking aromatic rings structures, while hemicellulose and cellulose are not damaged (Nakamura et al. 2004). It can be used to disrupt the structure of many different lignocellulosic materials, such as wheat straw, bagasse, pine, peanut, cotton straw and poplar sawdust (Sun and Cheng 2002). García-Cubero et al. (2009) investigated the pretreatment of wheat and rye straws with ozone to increase the enzymatic hydrolysis extent of potentially fermentable sugars. Enzymatic hydrolysis yields of up to 88.6 and 57 % were obtained compared to 29 and 16 % in non-ozonated wheat and rye straw, respectively. Moisture content and type of biomass showed the most significant effects on ozonolysis. In addition, ozonolysis experiments in basic medium with sodium hydroxide showed a reduction in solubilization of lignin and reliable cellulose and hemicellulose degradation. Miura et al. (2012) also studied the effect of ozonolysis and Wet Disk Milling (WDM) on Japanese cedar (*Cryptomeria japonica*) to improve sugar production by enzymatic saccharification. When the moisture content reached more than 40 %, ozone consumption decreased, resulting in less delignification. Ozone treatment removed mainly lignin, but also small amounts of polysaccharides. The application of WDM following the ozone treatment further increased glucose and xylose yields of 68.8 and 43.2 %, respectively, but had no significant effect on mannose yield. Similar to this study, ozonolysis in combination with WDM was used for pretreatment of sugarcane bagasse and straw (Barros Rda et al. 2013). Under optimized conditions, for sugar cane bagasse, yields of glucose (89.7 %) and xylose (48.8 %) were obtained after 60 min ozonolysis followed by WDM (4 cycles and 1.2 min/g). In case of straw, glucose and xylose saccharification yields of 92.4 and 52.3 % were obtained after WDM (4 cycles and 0.2 min/g) followed by 60 min ozonolysis, indicating that bagasse and straw responded differently to the same process conditions.

Ozonolysis alone has been shown to be ineffective for removal of lignin and yields of reducing sugars. However, combination of ozonolysis with other pretreatment process such as wet disk milling has shown promising results. Reports have demonstrated that sugar yields up to 90 % can be obtained by combination of these processes (Barros Rda et al. 2013). A major disadvantage of ozonolysis is the high costs that are involved, which inhibits its use for large-scale applications.

### Pretreatment by wet oxidation

Wet oxidation involves oxygen or air in combination with water at elevated temperature and pressure (Varga et al. 2003). As the name implies, in this process, oxidation of organic matter in the presence of oxygen takes place. When the process takes place at low temperatures, hydrolysis of lignocellulose occurs. At high temperatures, oxidation of lignocellulose occurs with liberation of carbon dioxide and water. Wet oxidation process solubilizes hemicellulose and lignin is degraded into carbon dioxide, water, and carboxylic acids such as succinic acid, glycolic acid, formic acid, acetic acid, phenolic compounds (Bjerre et al. 1996). It was presented as an alternative to steam explosion which had become the most widely used pretreatment method (Palonen et al. 2004). Industrially, wet air oxidation processes have been used for the treatment of wastes with a high organic matter by oxidation of soluble or suspended materials using either an oxidizing agent such as hydrogen peroxide or oxygen in aqueous phase at high temperatures (180–200 °C) (Jorgensen et al. 2007). The effect of wet oxidation to enhance anaerobic biodegradability and methane yields from different biowastes (food waste, yard waste, and digested biowaste) by employing thermal wet oxidation was studied by Lissens et al. (2004b). Measured methane yields for raw yard waste, wet oxidized yard waste, raw food waste, and wet oxidized food waste were 345, 685, 536, and 571 mL of CH_4_/g of volatile suspended solids, respectively. Higher oxygen pressure during wet oxidation of digested biowaste considerably increased the total methane yield and digestion kinetics and permitted lignin utilization during a subsequent second digestion. The increase of the specific methane yield for the full-scale biogas plant by applying thermal wet oxidation was 35–40 %, showing that there is still a considerable amount of methane that can be harvested from anaerobic digested biowaste. Fox and Noike (2004) evaluated wet oxidation method for increasing methane fermentation of newspaper waste. Wet oxidation was carried out at 170, 190, and 210 °C, with a retention time of 1 h. The highest removal efficiencies of newspaper waste in terms of change in total Chemical Oxygen Demand (TCOD) and cellulose were achieved at 210 °C during which approximately 40 % TCOD and 69 % Cellulose were reduced, respectively. On the other hand, highest lignin removal efficiency was achieved at 190 °C in which approximately 65 % was removed. Batch methane fermentation tests were performed in 2-L glass bottles filled with the wet oxidized newspaper samples. Methane fermentation of newspaper pretreated at 190 °C gave the highest methane conversion efficiency (59 % of the initial TCOD was recovered as methane gas). Similarly, pretreatment of organic municipal solid waste enriched with wheat straw by wet oxidation for subsequent enzymatic conversion and fermentation into bioethanol was investigated by Lissens et al. (2004a). The effect of temperature (185–195 °C), oxygen pressure (3–12 bar) and sodium carbonate (0–2 g) addition on enzymatic cellulose and hemicellulose convertibility was studied at a constant wet oxidation retention time of 10 min. An enzyme convertibility assay at high enzyme loading showed that up to 78 % of the cellulose and up to 68 % of the hemicellulose in the treated waste could be converted into, respectively, hexose and pentose sugars compared to 46 % for cellulose and 36 % for hemicellulose in the raw waste. For all wet oxidation conditions tested, total carbohydrate recoveries were high (>89 %) and 44–66 % of the original lignin could be converted into non-toxic carboxylic acids. Simultaneous Saccharification and Fermentation (SSF) of the treated waste by *Saccharomyces cerevisiae* yielded average ethanol concentrations of 16.5–22 g/L for enzyme loadings of 5 and 25 FPU/g, respectively. The cellulose to ethanol conversion efficiency during SSF was 50, 62, 65 and 70 % for a total enzyme loading of 5, 10, 15 and 25 FPU/g of biomass, respectively. Pretreatment of rice husk by wet air oxidation for the production of ethanol was studied by Banerjee et al. (2009). Optimum conditions were found to be of 185 °C, 0.5 MPa, and 15 min which yielded 67 % (w/w) cellulose content in the solid fraction, along with 89 % lignin removal, and 70 % hemicellulose solubilization. Szijártó et al. (2009) studied the treatment of a common reed (*Phragmites australis*) by employing wet oxidation to enhance the enzymatic digestibility of reed cellulose to soluble sugars, thus improving the convertibility of reed to ethanol. The most effective treatment increased the digestibility of reed cellulose by cellulases more than three times compared to the untreated control. During this wet oxidation, 51.7 % of the hemicellulose and 58.3 % of the lignin was solubilized, whereas 87.1 % of the cellulose remained in the solids. After enzymatic hydrolysis of pretreated fibers from the same treatment, the conversion of cellulose to glucose was 82.4 %. Simultaneous saccharification and fermentation of pretreated solids resulted in a final ethanol concentration as high as 8.7 g/L, yielding 73 % of the theoretical yield. Banerjee et al. (2011) investigated pretreatment of rice husk by Alkaline Peroxide-Assisted Wet Air Oxidation (APAWAO) to increase the enzymatic convertibility of cellulose in pretreated rice husk. Rice husk was presoaked overnight in 1 % (w/v) H_2_O_2_ solution at room temperature, followed by Wet Air Oxidation (WAO). APAWAO pretreatment resulted in solubilization of 67 wt% of hemicellulose and 88 wt% of lignin initially present in raw rice husk. APAWAO pretreatment resulted in 13-fold increase in the amount of glucose that could be obtained from otherwise untreated rice husk. Up to 86 % of cellulose in the pretreated rice husk could be converted into glucose within 24 h, yielding over 21 % glucose. Wet oxidation is considered a suitable process for pretreatment of biomass having high lignin content.

This survey indicates that with a variety of biomasses, this process has shown promising results. Reducing sugar yields up to 70 % have been obtained by utilizing this pretreatment process (Banerjee et al. 2009). The main drawback of the process is that it requires the maintenance of high temperature, pressure and the presence of strong oxidizing agents such as hydrogen peroxide. These requirements lead to high costs to maintain such conditions and also require large-scale reaction vessels to withstand such harsh conditions. Therefore, application of this process in large-scale pretreatment of biomass is limited.

## Pretreatment of biomass by biological methods

During conversion of lignocellulosic biomass, the pretreatment step is considered as rate limiting step and governs the final cost of products. Conventional physicochemical methods for lignin degradation require large inputs of energy and also cause pollution. Therefore, biological pretreatment of lignocellulosic biomass is considered as an efficient, ecofriendly and cheap alternative (Wan and Li 2012). The biological pretreatment of lignocellulosic biomass is usually performed by employing cellulolytic microorganisms which synthesize potent cellulolytic enzymes during hydrolysis. The common candidates are filamentous fungi, which are ubiquitous in the environment such as soil, living plants and lignocellulosic waste material. Studies have shown that white-rot fungi are the most effective microorganisms for the pretreatment of lignocelluloses such as wood chips, wheat straw, Bermuda grass and softwood (Akin et al. 1995). These fungi are capable of degrading cellulose, hemicellulose and lignin and are considered as major degraders of woods in forest ecosystems. Another class of fungi, commonly known as brown-rot fungi can selectively degrade cellulose and hemicellulose, without affecting lignin (Rasmussen et al. 2010). The mechanism of degradation of lignocellulose by fungi is fully understood and can be broadly divided into two main categories: oxidative and hydrolytic. In oxidative mechanism, lignin is degraded via production of Reactive Oxygen Species (ROS) mainly hydroxyl radicals by the fungi (Hammel et al. 2002). Many fungi are known to produce hydrogen peroxide by the action of enzymes such as glyoxal oxidase, pyranose-2 oxidase, and aryl-alcohol oxidase, respectively (Martinez et al. 2009). Hydrogen peroxide then reacts with iron via the Fenton reaction to produce hydroxyl radicals. These radicals degrade lignin and produce low molecular weight products (Hammel et al. 2002). Another group of enzymes that degrade lignin are laccases and manganese peroxidase (MnP). Laccase is a multicopper oxidase and can catalyze free radicals-mediated reactions that degrade lignin (Eggert et al. 1997). Manganese peroxidase on the other hand oxidizes Mn^2+^ into Mn^3+^ by hydrolysis of hydrogen peroxide (Hofrichter 2002). Mn^3+^ is a powerful oxidant that degrades lignin. In hydrolytic mechanism, the fungi produce hydrolytic enzymes which degrade glycosidic linkages in cellulose and hemicellulose releasing monomeric sugars (Feijoo et al. 2008). Cellulose degradation is achieved by synergistic action of three classes of hydrolytic enzymes: endo-(1,4)-β-glucanase (endocellulase), cellobiohydrolase (exocellulase), and β-glucosidase (Baldrian and Valaskova 2008). Whereas, hemicellulose degradation is achieved by action of hydrolytic enzymes such as endo-xylanases, endo-α-l-arabinase, endo-mannanase, β-galactosidase, and β-glucosidases, respectively (Shallom and Shoham 2003). By synergistic action of both these pathways, lignocellulose is degraded by fungi. Biological pretreatment of wheat straw by white-rot fungus Euc-1 was performed by Dias et al. (2010). The effect of fungal pretreatment on the cellulose accessibility toward commercial cellulolytic enzyme preparation (Onozuka R-10) has been evaluated. Results indicated that after 23 days of treatment following enzyme treatment, maximum reducing sugar yield of 23 % was observed. Also 7 % decrease in total lignin content was also recorded at these treatment conditions. Biological pretreatment of different feed stocks such as corn stover, wheat straw, soybean straw, switch grass, and hardwood by *Ceriporiopsis subvermispora* was evaluated by (Wan and Li 2011). Results of their study indicated that after 18-day pretreatment, corn stover, switch grass, and hardwood were effectively delignified by the fungus by the combined action of manganese peroxidase and laccase. Glucose yields during enzymatic hydrolysis reached 56.5, 37.2, and 24.2 %, respectively, which were a 2- to 3-fold increase over those of the raw materials. A further 10–30 % increase in glucose yields was observed when pretreatment time extended to 35 days. Biological pretreatment of rice husks by a white-rot fungus *Phanerochete chrysosporium* is performed in one step consisting of biological and enzymatic treatment, respectively (Potumarthi et al. 2013). Results demonstrated that fungal pretreated rice husk produced highest 44.7 % reducing sugars on the 18th day of fungal treatment. Similarly, Yu et al. (2010) evaluated the effects of biological treatment using *Ganoderma lucidum*, *Trametes versicolor* and *Echinodontium taxodii* prior to alkaline/oxidative (A/O) pretreatment. Results showed that *Echinodontium taxodii* significantly enhanced the efficiency of chemical pretreatment. Subsequent to treatment of corn straw with *Echinodontium taxodii* for 15 days, the straw was subjected to digestion by 0.0016 % NaOH and 3 % H_2_O_2_ at room temperature for 24 h, which increased the reducing sugar yield by 50.7 %. Biological treatment of wheat straw by solid state and submerged fermentations in the presence of white-rot basidiomycetes such as *Bjerkandera adusta*, *Fomes fomentarius*, *Ganoderma resinaceum*, *Irpex lacteus*, *Phanerochaete chrysosporium*, *Trametes versicolor*, Euc-1, and *Lepista nuda* were evaluated by Pinto et al. (2012). Results of their study indicated that enzymatic hydrolysis of holocellulose after solid-state pretreatment showed that treatment with *T. versicolor* leads to a significant increase of saccharification process. The total reducing sugar yield was 7 %, which was 91 % higher as compared to untreated straw. López-Abelairas et al. (2012) studied the biological pretreatment of lignocellulosic biomass for the production of bioethanol using ligninolytic fungi (*Pleurotus eryngii* and *Irpex lacteus*) in a solid-state fermentation of sterilized wheat straw complemented with a mild alkali treatment. The largest digestibility was achieved with fermentation with *I. lacteus* under optimized conditions, under which cellulose and hemicellulose digestibility increased after 21 days of pretreatment from 16 to 100 % and 12 to 87 %, respectively. The maximum glucose yield (84 %) of cellulose available in raw material was obtained after only 14 days of pretreatment with an overall ethanol yield of 74 % of the theoretical value, which is similar to that reached with SE. Saritha et al. (2012) studied the treatment of hardwood and softwood by employing *Streptomyces griseus* isolated from leaf litter and showed that it enhanced the mild alkaline solubilization of lignin and also produced high levels of the cellulase when growing on wood substrates. Lignin loss (Klason lignin) observed was 10.5 and 23.5 % in case of soft wood and hard wood, respectively. Thus, biological pretreatment process for lignocellulosic substrate using lignolytic organisms such as actinomycetes and white-rot fungi was found to be efficient and cost effective. Performing saccharification and fermentation processes at high-substrate concentration has shown that it increases the concentration of inhibitors such as furan derivatives and phenolic compounds, which eventually inhibit the fermentation process. To inhibit production of inhibitors, treatment with enzymes such as laccases has been suggested. Alvira et al. (2012) studied the treatment of laccase on concentration of phenolic compounds. The results demonstrated that supplementation of laccase reduced the phenolic content and increased the performance of *Saccharomyces cerevisiae* during fermentation. This survey clearly suggests that total reducing sugar in yield in biological treatment of biomass ranges from 30 to 60 %, after 10–30 days of incubation (Wan and Li 2011). Degradation of lignin and extraction of phenolic compounds from the leaves of *Larrea tridentata* by a combination of biological treatment with basidiomycete *Phanerochaete chrysosporium* followed by organosolv extraction by methanol were evaluated by Martins et al. (2013). Results clearly indicated that recovery of phenolic compounds was 33 % more with combination of biological treatment followed by methanol extraction as compared to methanol extraction alone.

Pretreatment of biomass with fungal and bacterial strains for cellulose hydrolysis is inexpensive and easy to operate. Biological pretreatment of biomass is considered to be an inexpensive process when compared to other pretreatment processes such as organosolv or AFEX, where the cost of chemicals and infrastructure is high (Hasunuma et al. 2013). However, large-scale operation of the process leads to higher operational costs. To carry out biological pretreatment of biomass on a large scale, a large, sterile area is required and it is necessary to maintain the sterile conditions during the treatment process. This important requirement increases the cost of biological pretreatment process. The second key disadvantage of this process is that a major portion of carbohydrates such as cellulose and hemicellulose are consumed by the microorganisms, i.e., fungi. This leads to lower outputs and increases the cost of treatment process. Furthermore, the process is too slow and thus it is not recommended for industrial purposes.

## Pretreatment of biomass by microwave irradiation

Microwaves are radio waves with wavelengths ranging from 1 m to 1 mm, or equivalently, with frequencies between 300 MHz (0.3 GHz) and 300 GHz. When these waves interact with organic matter, they get absorbed by water, fats and sugars and their energy gets transferred to organic molecules generating enormous amount of heat. In this way, microwaves exhibit heating effect (Banik et al. 2003). Microwaves are also being employed in the treatment of lignocellulosic biomass (Ooshima et al. 1984). Studies have shown that microwaves cause localized heating of biomass leading to disruption of lignocellulose architecture. Thus, cellulose and hemicellulose get accessible to enzymatic hydrolysis (Sarkar et al. 2012). Microwave pretreatment of rice straw was studied by Maa et al. (2009). In their study, Box–Behnken design and response surface methodology were used to optimize the microwave pretreatment of rice straw. Under optimized conditions consisting of microwave intensity 680 W, irradiation time 24 min and substrate concentration 75 g/L, maximal efficiencies of cellulose, hemicellulose and total saccharification were, respectively, increased by 30.6, 43.3 and 30.3 %. In a similar study, Beech wood powder was treated with H_2_O_2_ and nine different ammonium salts (sulfate, chloride, hydrogen carbonate, nitrate, hydrogen phosphate, tartrate, carbonate, molybdate and acetate) at 80 °C for 5 h in an autoclave, and enzymatic saccharification of the resultant pulp was carried out using cellulolytic enzymes from *T. viride* (Verma et al. 2011). Ammonium molybdate (50 mM) gave the highest sugar yield of 50.1 %. The same process was repeated with microwave treatment. Under optimized conditions, the maximum sugar yield of 59.5 % was obtained by microwave irradiation at 140 °C for 30 min with ammonium molybdate and H_2_O_2_. Su et al. (2010) studied the effects of microwave treatment on sorghum liquor waste for bioethanol production. Their results indicated that reducing sugar yield following microwave treatment was tremendously high as compared to untreated waste. Microwave-assisted pretreatment of recalcitrant softwood in the presence of aqueous glycerol and different organic and inorganic acids was studied by Liu et al. (2010). Their study indicated that pulp obtained by organosolv lysis with 0.1 % hydrochloric acid (p*K*_a_ −6) at 180 °C for 6 min gave the highest sugar yield, 53.1 %. With other acids such as phosphoric acid, malonic acid having lower p*K*_a_ value, a lower sugar yield was obtained. These results clearly indicate that microwave-assisted glycerolysis is a suitable process for treatment of a variety of soft woods.

Microwave pretreatment of wheat straw for ethanol production was studied by Xu et al. (2011). In their study, an orthogonal design was used to optimize the microwave pretreatment on wheat straw for ethanol production. According to the orthogonal analysis, pretreatment with the ratio of biomass to liquid at 80 g/kg, the NaOH concentration of 10 kg^3^, the microwave power of 1,000 W for 15 min was confirmed to be the optimal condition. The ethanol yield was 14.8 and was much higher from untreated material which was only 2.6 %. Boonmanumsin et al. (2012) studied the microwave-assisted ammonium hydroxide treatment of *Miscanthus sinensis*. The results indicated that yield of monomeric sugars was substantially increased in the presence of microwave treatment. In a new approach, microwave pretreatment of oil palm empty fruit bunch fiber in the presence of alkaline conditions was studied by Nomanbhay et al. (2013). Their study showed that at optimum conditions of pretreatment, 3 % (w/v) NaOH at 180 W for 12 min led to the loss of 74 % lignin and 24.5 % holocellulose. The yield of total reducing sugars was 41 %.

Microwave treatment of biomass is considered a harsh process which leads to high lignin removal and increased sugar yields. This survey indicates that the yield of reducing sugars ranges from 40 to 60 % (Verma et al. 2011). These results have clearly indicated that microwave pretreatment of biomass gives promising results. But when efficacy of this process in terms of cost is considered, the process is very costly (Feng and Chen 2008). For large-scale pretreatment of biomass, a large microwave irradiator is required, which is costly, energy consuming and limits its use in large-scale operations. Another major disadvantage of the process is generation of high temperature and non-uniform heating of biomass, which leads to the formation of inhibitors and thus, the yields are generally lower as expected. This drawback also increases the operational cost (Jackowiak et al. 2011).

A summary of major treatment processes and a comparison between the yields of reducing sugars obtained are summarized in Table 1. It is clear from the table that none of the process discussed are efficient enough to give 100 % yield of reducing sugars from all types of biomasses. The efficiency of the process depends mainly on the type of biomass used as raw material, its structure and lignin content (Mckendry 2002; Cao et al. 2012). One major point that emerges from this discussion is that combination of two or more pretreatment processes is proven to be efficient when compared with single pretreatment process alone in terms of reducing sugar yield and lignin removal from different biomasses. Therefore, combinations of pretreatment processes are being tested for increasing the yield of reducing sugars (Cara et al. 2006; Yu et al. 2009; Miura et al. 2012).

**Table 1:** Summary of various pretreatment processes, their advantages/disadvantages, pretreatment conditions and percent yield

Pretreatment process	Conditions	Advantage	Disadvantage	Biomass/treatment	Yield (%)	Reference
Acid treatment	Dilute/concentrated acid/few minutes/high and low temperature	Increase in porosity/increased enzymatic hydrolysis	Synthesis of furfural/hydroxymethyl furfural/need for recycling/costly	Olive tree biomass/170 °C and 1.0 % acid	48.6 % Reducing sugar	Cara et al. (2008)
				Corn fibers/0.5 % sulfuric acid, 5 % biomass and at 140 °C	56.8 % Reducing sugar	Noureddini and Byun (2010)
				Sugarcane tops/25 % w/w biomass, 3 % sulfuric acid for 60 min	68.5 % Reducing sugar	Sindhu et al. (2011)
Alkaline treatment	Alkali treatment at NTP	Removal of lignin/hemicellulose hydrolysis	Formation of salts of calcium and magnesium	Corn stover/0.5 g Ca(OH)_2_/g, 55 °C for 4 weeks with aeration	91.3 % Glucose, 51.85 % xylose	Kim and Holtzapple (2005)
				Spruce/3 % NaOH/12 % urea and −15 °C	60 % Glucose	Zhao et al. (2008)
				Switch grass/0.5 % KOH, at 21 °C and after 12 h treatment	58.2 % Reducing sugar	Sharma et al. (2012)
Ammonia treatment	Ammonia at elevated temperatures	Removal of lignin/decrystallizing cellulose	Removal of ammonia/costly	Corn stover/first stage 190 °C, 5.0 mL/min, 30 min for hot water treatment and 170 °C, 5.0 mL/min, 60 min for aqueous ammonia treatment	78 % Glucan, 3.6 % xylan	Kim and Lee (2006)
				Barley hull/15–30 % aqueous ammonia, at 30–75 °C for 12 h to 77 days	83 % Glucan, 63 % xylan	Kim et al. (2008)
AFEX	Liquid ammonia at high temperature and pressure	Lignin removal/hydrolysis of hemicellulose/decrystallization of cellulose	Costly/not employed for high lignin content	Canary grass/100 °C, 60 % moisture content, 1.2:1 kg ammonia/kg of dry matter	86 % Glucose, 78 % xylose	Bradshaw et al. (2007)
				Empty palm fruit bunch fiber/135 °C, 45 min retention time, water to dry biomass loading of 1:1 (weight ratio), and ammonia to dry biomass loading of 1:1 (weight ratio)	90 % Reducing sugar	Lau et al. (2010)
Organosolv	Organic solvents at both high and low temperatures	Pure lignin obtained and used as value added product	Solvents inhibit enzymatic hydrolysis/costly	Wheat straw/glycerol, 20 g/g at 220 °C for 3 h	90 % Enzymatic hydrolysis	Sun and Chen (2008)
				Sugarcane bagasse/30 % (v/v) ethanol at 195 °C, for 60 min	29.1 % Reducing sugars	Mesa et al. (2011)
				Oil palm pulp/ethylene glycol–water	50.1 % Reducing sugars	Ichwan and Son (2011)
Oxidative delignification	Treatment with oxidizing agents	Fast process	Formation of acids which act as enzyme inhibitors/costly/hemicellulose degradation	Rice hull/H_2_O_2_ (2 %, 48 h) and *P. ostreatus* (18 days)	39.8 % Reducing sugar	Yu et al. (2009)
				Sweet sorghum bagasse/dilute NaOH, autoclaving and H_2_O_2_	90.9 % Reducing sugar	Cao et al. (2012)
				Cashew apple bagasse/5 % (w/v) at 4.3 % AHP, 6 h, 35 °C	42.9 % Reducing sugar	Correia et al. (2013)
Ozonolysis	Treatment with ozone	Lignin is damaged/cellulose/hemicellulose unaltered	Costly	Japanese cedar/of ozonolysis and wet disk milling	68.8 % Glucose, 43.2 % xylose	Miura et al. (2012)
				Straw/wet disk milling (4 cycles and 0.2 min/g) followed by 60 min ozonolysis	92.4 % glucose, 52.3 % xylose	Barros Rda et al. (2013)
Wet oxidation	Oxidation at elevated temperature	Treatment of wastes	Costly	Wheat straw/195 °C, 2 g sodium carbonate	78 % Cellulose and 68 % hemicellulose converted into hexoses	Lissens et al. (2004a)
				Rice husk/185 °C, 0.5 MPa and 15 min	70 % Hemicellulose solubilization	Banerjee et al. (2009)
				Rice husk/alkaline peroxide-assisted wet air oxidation	21 % Glucose	Banerjee et al. (2011)
Biological	Treatment with various lignocellulolytic microbes	Cheap/ecofriendly	A part of fermentable sugars are utilized as carbon source/slow process	Wheat straw/white-rot fungus Euc-1, 23 days treatment	23 % Reducing sugar	Dias et al. (2010)
				Corn stover/*Ceriporiopsis subvermispora,* 18 days	56.5 % Glucose	Wan and Li (2011)
				Rice husk/white-rot fungus *Phanerochete chrysosporium*	44.7 % Reducing sugar	Potumarthi et al. (2013)
Microwave	Treatment with microwaves	Cheap/generated less pollution	Degradation of cellulose/hemicellulose	Rice straw/microwave intensity 680 W, irradiation time 24 min and substrate concentration 75 g/L	30.3 % Total sugar	Maa et al. (2009).
				Beech wood/microwave irradiation at 140 °C for 30 min with ammonium molybdate and H_2_O_2_	59.5 % Total sugar	Verma et al. (2011)
				Soft wood/0.1 % HCl (p*K*_a_ −6) at 180 °C for 6 min	53.1 % Total sugar yield	Liu et al. (2010)

## Conclusion

The impact of green house gases on the climate change has been recognized as a serious environmental threat. Efforts are being made for the search of more efficient, sustainable and environmental friendly technology to prevent such emission. Due to immense potential for conversion of renewable biomaterials into biofuels, production of ethanol from lignocellulosics has received much attention since the last decade. A major bottleneck in this technology is the presence of lignin, which is a major inhibitor of hydrolysis of cellulose and hemicellulose. This has led to extensive research in the development of various pretreatment processes for the treatment of lignocellulosic biomass. These processes were based on physical, chemical and biological principles. We have discussed various pretreatment processes that are commonly employed in the treatment of biomass. The major advantages, disadvantages and yield of pretreatment processes have been discussed. One important point that emerges is that no treatment technology offers 100 % conversion of biomass into fermentable sugars. There is always a loss of biomass, which affects the final yield and increases the cost of finished product, i.e., biofuel. Although pretreatment of lignocellulosic biomass with combination of two or more pretreatment processes have shown promising results, we still feel that there is a need for extensive research in this area, so that either a new efficient treatment process is developed or an existing process is upgraded to give promising results.
